# Research-Tested Mobile Apps for Breast Cancer Care: Systematic Review

**DOI:** 10.2196/10930

**Published:** 2019-02-11

**Authors:** Chiara Jongerius, Selena Russo, Ketti Mazzocco, Gabriella Pravettoni

**Affiliations:** 1 Department of Medical Psychology Amsterdam UMC University of Amsterdam Amsterdam Netherlands; 2 Kids Cancer Centre Sydney Children's Hospital University of New South Wales Sydney Australia; 3 Department of Oncology and Hemato-oncology IEO, European Institute of Oncology IRCCS Milan Italy

**Keywords:** breast cancer care, breast cancer management, breast cancer prevention, breast cancer survivorship, mobile applications, mHealth applications

## Abstract

**Background:**

The use of mobile health (mHealth) apps in clinical settings is increasing widely. mHealth has been used to promote prevention, improve early detection, manage care, and support survivors and chronic patients. However, data on the efficacy and utility of mHealth apps are limited.

**Objective:**

The main objective of this review was to provide an overview of the available research-tested interventions using mHealth apps and their impact on breast cancer care.

**Methods:**

A systematic search of Medline, PsycINFO, Embase, and Scopus was performed to identify relevant studies. From the selected studies, the following information was extracted: authors, publication date, study objectives, study population, study design, interventions’ features, outcome measures, and results.

**Results:**

We identified 29 empirical studies that described a health care intervention using an mHealth app in breast cancer care. Of these, 7 studies were about the use of an mHealth application in an intervention for breast cancer prevention and early detection, 12 targeted care management, and 10 focused on breast cancer survivors.

**Conclusions:**

Our results indicate consistent and promising findings of interventions using mHealth apps that target care management in breast cancer. Among the categories of mHealth apps focusing on survivorship, mHealth-based interventions showed a positive effect by promoting weight loss, improving the quality of life, and decreasing stress. There is conflicting and less conclusive data on the effect of mHealth apps on psychological dimensions. We advocate further investigation to confirm and strengthen these findings. No consistent evidence for the impact of interventions using mHealth apps in breast cancer prevention and early detection was identified due to the limited number of studies identified by our search. Future research should continue to explore the impact of mHealth apps on breast cancer care to build on these initial recommendations.

## Introduction

Mobile health (mHealth) is a method to deliver health care or related services through portable devices [1] and is broadly accessible and often freely delivered in the app stores of app providers. The online app market is open to developers and allows them to sell or provide their apps free of charge. The current estimates suggest that there are more than 40,000 mHealth apps [2]. According to a recent report by Grand View Research, Inc., the global mHealth market is expected to reach US $111.8 billion by 2025, and there is a growing need to reduce long waiting periods to access health care services, which is the primary driver responsible for the adoption of mHealth [3].

mHealth apps are extremely relevant for both industrialized nations and developing countries, as they provide extended access to health care and health-pertinent information in a cost-effective way [4]. Scholars and health care professionals have shown interest in this new technology, and the use of mHealth in clinical settings is increasing widely [5]. Recent reviews showed that in cancer care, mHealth apps have been employed to promote prevention, improve early detection, manage cancer care, and support cancer survivors [5,6]. Furthermore, research-tested apps offer the unique possibility of providing accessible information and education at minimal costs throughout the cancer care continuum [5].

Despite their impressive and promising potential, the utility and effectiveness of this e-health technology remain unclear. Scholars have reviewed the use of mHealth apps in several fields [4,7-10]. Mobasheri et al [4] reviewed 158 mHealth apps for breast cancer and found that there is a lack of evidence on their utility, effectiveness, and safety. Most mHealth apps lack expert involvement and do not adhere to relevant medical evidence or reflect patients’ needs [11]. In addition, given the large number of available mHealth apps, it seems unrealistic to test each app thoroughly and scientifically before its release [4,5]. A recently developed mHealth app had negative effects on patients, augmenting their anxiety after breast cancer surgery [12]. The mHealth app aimed at providing extensive information to patients; however, the control patients, who did not use the application, had lower anxiety levels than the test patients, which correlated to higher quality of life in the former group [12].

The possibilities to enhance positive health outcomes and promote patients’ feelings of control over their health through the use of mHealth technology should be fully explored and tailored to patients’ needs [13-15]. Scholars and stakeholders advocate for more medical professional involvement, inclusion of patients’ preferences, and specific regulations [4].

The need for integration between research-tested and privately developed mHealth apps has been widely stressed, as such integration plays a crucial role in achieving the necessary fundamentals of effective, evidence-based care [10,16,17]. Research should provide a scientific basis to build elements that can be effective for patient care. It is especially important to know which mHealth app has been scientifically tested and provides an overview of the interventions using mHealth apps. Our focus is on mHealth apps in breast cancer care. The present review aimed to provide an overview of the available evidence on research-tested interventions using mHealth apps in breast cancer care. Our findings aim to provide valuable information to health care professionals, mHealth apps developers, breast cancer patients, and other stakeholders on the characteristics of existing research-tested apps used in breast cancer care, including their advantages and pitfalls, in order to correctly, effectively, and safely support breast cancer patients.

## Methods

### Search Strategy and Selection of Articles

An extended bibliographic search was conducted in the Medline, PsycINFO, Embase, and Scopus databases. The following search string was used: ((phone OR mobile OR smartphone*) AND (app* OR application*)) AND ((breast* OR mammary) AND (cancer* OR neoplasm* OR carcinom* OR tumor*)).

The search was limited to studies published from January 2008 (when the first mobile phone app was created) to September 2018. The review was registered in PROSPERO with the registration number CRD42017056239. All detected articles were screened according to the following inclusion criteria: original studies, English language, studies that include the use of an mHealth app, studies that include patients’ use of an mHealth app, and studies that concern the prevention/detection/care management/survivorship of breast cancer. We excluded studies that used websites, text messaging, emails, or other technological interventions that did not include mobile apps and studies that used mobile apps without applying them to breast cancer patients (eg, health care professionals that use mHealth apps).

### Data Collection and Extraction Process

A data-extraction form was developed on basis of the Centre for Reviews and Dissemination templates [18]. Two reviewers independently extracted the data from the included studies by using the extraction form. Disagreements in data extraction were resolved through discussions between the authors until an agreement was reached. Relevant articles were then selected by cross-examining and reviewing the articles. Data collected included information on authors, publication date, intervention’s features, study design, sample size, outcome measures, and results. The quality of the quantitative studies was evaluated independently by two researchers using the Effective Public Health Practice Project Quality Assessment Tool for Quantitative Studies [19]. This tool provides a standardized means to assess the quality of a quantitative study, leading to an overall methodological rating of strong, moderate, or weak in eight sections: selection bias, study design, confounders, blinding, data-collection methods, withdrawals and dropouts, intervention integrity, and analysis. The quality of the qualitative studies was evaluated using the Joanna Briggs Institute Critical Appraisal Checklist for Qualitative Research [20]. This instrument is underpinned by a multi-dimensional concept of quality in research, and the 10 items assess quality according to several domains including quality of reporting, methodological rigor, and conceptual depth and bread. Discordances in quality rating were resolved through discussion between the researchers.

## Results

### Study Selection

**Figure 1:**
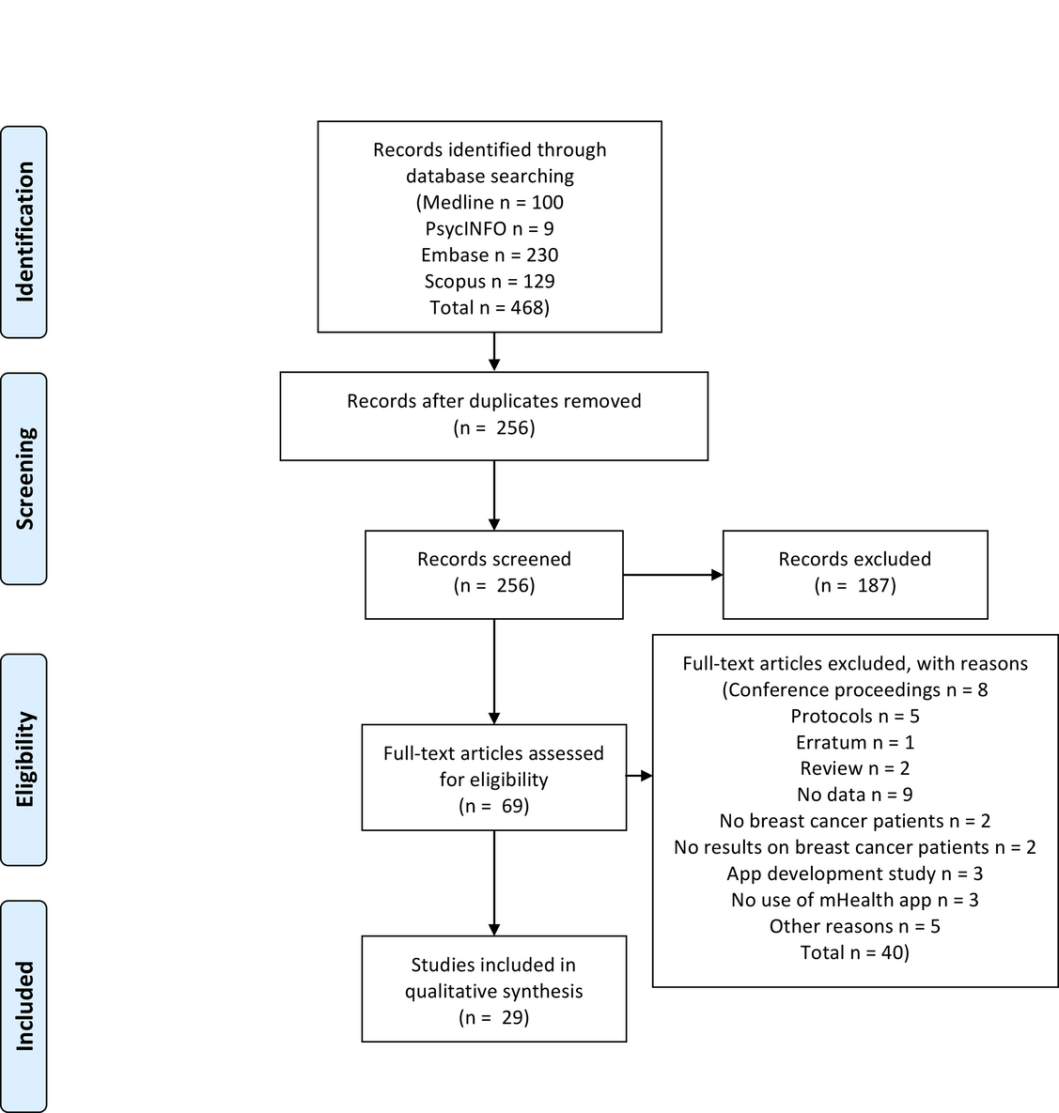
Flow diagram of identification, screening, eligibility, and inclusion of studies.

The results of the systematic search are summarized in Figure 1 in accordance with the Preferred Reporting Items for Systematic Reviews and Meta-Analyses [21]. We identified a total of 256 articles (100 from Medline, 9 from PsycINFO, 230 from Embase, and 129 from Scopus), from which we excluded 212 duplicates. Abstracts and titles were screened to identify articles discussing an intervention using an mHealth app for the care of patients with breast cancer, and 69 such articles were found. Further screening on the basis of the entire text according to our inclusion and exclusion criteria led to a final selection of 29 articles.

### Study Characteristics

The 29 studies identified were published between 2011 and 2018. Majority of the studies (n=12) focused on apps for breast cancer care management, 10 focused on survivorship, and 7 focused on prevention and early detection.

Ten of the 29 studies were randomized controlled trials, 8 were prospective cohort studies, 7 were cross-sectional studies, and 4 used qualitative analysis. Using the Quality Assessment Tool for Quantitative Studies [19], 10 studies were rated as strong; 9, as moderate; and 6, as weak. The 4 qualitative studies assessed using the Critical Appraisal Checklist for Qualitative Research [20] revealed a methodological quality that allowed their inclusion in the review. Two of the 29 studies focused on the use of an mHealth app in breast cancer prevention, 5 targeted early detection, 12 were on care management, and 10 focused on survivors of breast cancer. The sample included in the studies comprised adult patients in different countries with different ethnic backgrounds: 14 studies in North America, 8 in Europe, and 6 in Asia. Table 1 provides a summary of the features of the included studies.

**Table 1:** Description of the characteristics of the studies presented in the review^a^.

Characteristics and studies		Intervention target	Sample size	Duration & follow-up	Intervention components	Outcome measures	Study quality^b^
**Prevention**							
	Alanzi et al [23] (2018)	To create awareness about breast cancer	Intervention group: N=96; Control group: N=95	4 weeks; Measurements at baseline and at 4-week follow-up	*SnapChat* app: breast cancer awareness information on mobiles, covering knowledge about breast cancer, its symptoms, diagnosis process, and available treatments. New information provided three times each week	18-item questionnaire on breast cancer awareness	Moderate
	Hartman et al [22] (2016)	Weight loss	Intervention group: N=36; Usual care group: N=18	6 months	Combined technology-based self-monitoring tools with individualized phone calls: Electronic calorie-counting tool (*MyFitnessPal*); 12 phone calls (30 minutes each) over 6 months; Accelerometer-based activity meter that provides real-time feedback on the number of steps taken and minutes of moderate-intensity activity (*Fitbit*)	Weight and accelerometer-measured physical activity	Strong
**Early detection**							
	Eden et al [24] (2015)	To help women in their 40s gain deeper insights into their priorities for screening and prepare them to discuss mammography screening with their health care providers	N=75	Before and after use of the app (same day)	The decision aid (*Mammopad*) included educational modules on breast cancer, mammography, risk assessment, and priority setting about screening	Decisional conflict; Decision self-efficacy scale	Moderate
	Heo et al [25] (2013)	To encourage breast self-examination	N=45	Before and after use of the app	A mobile phone app developed with functions including a breast self-examination date alarm, a reminder to encourage mother and daughter to practice breast self-examination together, record keeping, and educational content with video clips	Survey: increased breast self-examination	Weak
	Keohane et al [26] (2017)	To improve risk perception	Intervention group: N=42; Usual care group: N=42	Measurements before the counseling session (T1), after the counseling session (T2), and 6 weeks later (T3)	A mobile phone app displaying data on risk of developing breast cancer as well as risk of carrying the *BRCA* gene	IBIS^c^ Breast Cancer Risk Evaluation; Perception of risk: “Patient Survey on Risk Perception of Breast Cancer and Health Literacy”	Strong
	Lee et al [27] (2017)	To increase knowledge and awareness and promote mammogram screening	Intervention group N=60 Usual care group N=60	1 week; Measurements at baseline, 1 week, and 6 months	*mMammogram*: Each day participants received 8-21 messages covering various topical areas, including breast cancer, screening guidelines, and types of screening; breast cancer risk factors; individual, structural, and cultural barriers to screening; communication strategies; follow-up for test results; and information on local clinics. Messages followed a trajectory from basic knowledge building to specific strategies aimed to enhance motivation for and access to mammography	Knowledge, attitudes, and beliefs about breast cancer screening, readiness for mammography, and mammogram receipt; Feasibility and acceptability of the mMammogram intervention	Strong
**Care management**							
	Lee et al [28] (2018)	To increase knowledge and awareness and promote mammogram screening	N=14	1 week	*mMammogram*: Each day participants received 8-21 messages covering various topical areas including breast cancer, screening guidelines, and types of screening; breast cancer risk factors; individual, structural, and cultural barriers to screening; communication strategies; follow-up for test results; and information on local clinics. Messages followed a trajectory from basic knowledge building to specific strategies aimed to enhance motivation for and access to mammography	Thematic analysis was used to analyze data from focus groups	Included
	Egbring et al [29] (2016)	Collection of patient-reported daily functional activity	Control group: N=41; Unsupervised intervention group: N=45; Supervised intervention group: N=41	3 visits	Mobile and Web app to record daily functional activity and adverse events: Patients could report daily functional activity or symptoms with indication of severity	Functional activity; Eastern Cooperative Oncology Group scoring; Common Terminology Criteria for Adverse Events	Strong
	Foley et al [12] (2016)	To decrease anxiety levels of patients undergoing surgery through educational materials	Intervention group: N=13; Control group: N=26	Measurement one day before, one day after, and 7 days after surgery	An iPad app containing tailored information on surgery pertaining to individual patients	Mini-Mental Adjustment to Cancer questionnaire; Hospital Anxiety and Depression Scale; Information Satisfaction Questionnaire	Moderate
	Harder et al [39] (2017)	To optimize self-management of arm and shoulder exercises for upper-limb dysfunction after breast cancer treatment	N=3	8 weeks; Measurement at the end of the study	*bWell* is an evidence-based program providing progressive exercises for passive and active mobilization, stretching, and strengthening. App features include tailored information, video demonstrations of the exercises, push notifications, and tracking and progress features	Questionnaire capturing users’ feedback and evaluating content, functionality (including ratings), and explored areas of improvement	Weak
	Hwang [30] (2016)	To communicate and share images of the wound postoperatively	Intervention group: N=35; Control group: N=37	1, 3, 7, and 14 days after surgery	A virtual care platform that consists of a mobile phone app and secure password-protected online account (*Medeo* app). Patients can take photos of their wounds postoperatively and send them to the surgeon using the mobile phone app. The surgeon then responded to each patient message within 24 hours.	Less readmission to the hospital; Use of mobile phone app for question; Improved perceived care	Moderate
	Kim et al [31] (2016)	To collect and track daily mental health indicators for depression	N=78	48 weeks	A mobile mental health tracker (part of *Pit-a-Pat* app) that uses three daily mental health ratings (sleep satisfaction, mood, and anxiety) as indicators for depression	Patient Health Questionnaire-9; Adherence level	Moderate
	Klasnja et al [34] (2011)	Manage care-related information	N=9	4 weeks; Measurement at baseline, 2 weeks, and 4 weeks	A web component (*HealthWeaver* website) to manage personal and health issues. It provides a calendar to manage health-related events; functionality for organizing notes, lists, bookmarks, and care-related files; a tracking system for symptoms, pain, and well-being; and logs for medications, supplements, and the care of post-surgery wounds. A mobile component (*HealthWeaver Mobile*) through which patients can create, edit, and view the information stored in the website; share them with family members and health care professionals; create photo, audio, and text notes and link those notes to related appointments; and synchronize the app calendar to the phone’s calendar app	Qualitative thematic analysis of interviews (at 2 weeks and 4 weeks) + demographics and experience with technology questionnaires (at baseline)	Included
	Min et al [32] (2014)	Sleep disturbance-related data collection from breast cancer patients receiving chemotherapy	N=30	90 days	App (*Pit-a-Pat*) developed to self-report three health experiences that may be caused by breast cancer diagnosis and treatments: sleep-disturbance symptoms related to mild depression, acute symptoms related to cytotoxic chemotherapeutic agents, and medication diary for antihormonal treatment	Compliance to use of mobile app	Moderate
	Rosen et al [37] (2018)	To decrease physical and psychological distress and improve the quality of life	Intervention group: N=57; Control group: N=55	8 weeks; Measurement at baseline, during intervention (5‐weeks), after intervention (9‐weeks), and follow‐up (12‐weeks)	Mindfulness meditation training was delivered through a commercially available mindfulness app (*Headspace*) that uses audio and animated video	The Brief Pain Inventory‐Short Form-32; The Brief Health Literacy Screening Tool; The eHealth Literacy Scale; Functional Assessment of Cancer Therapy—Breast version; Mindful Attention Awareness Scale; App utilization	Strong
	Wallwiener et al [38] (2017)	Tablet-based measurement app for EORTC QLQ-C30^d^	N=106	Not available	e-Patient-Reported Outcome versions of the EORTC QLQ-C30^d^ questionnaires	Electronic and paper-based versions of the health-related quality of life EORTC QLQ-C30^d^ questionnaire	Strong
	Weaver et al [33] (2014)	Real-time symptom monitoring of patients receiving oral chemotherapy	N=26	8 times for 3 weeks	Patients completed a symptom, temperature, and dose diary twice a day using a mobile phone app. This information was encrypted and automatically transmitted in real time to a secure server, with moderate levels of toxicity automatically prompting self-care symptom management messages on the screen of the patient’s mobile phone or in severe cases, a call from a specialist nurse to advice on care according to an agreed protocol	Medication dose and monitoring side effects	Weak
	Zhu et al [35] (2018)	To address women’s self-efficacy, social support, symptom distress, quality of life, anxiety, and depression	N=13	12 weeks; Measurements at the end of intervention	Breast Cancer e-Support with four components: a Learning forum, a Discussion forum, an Ask-the-Expert forum, and a Personal Stories forum.	Inductive content analysis of the interviews	Included
	Zhu et al [36] (2018)	To address women’s self-efficacy, social support, symptom distress, quality of life, anxiety, and depression	Intervention group: N=55; Control group: N=49	12 weeks; Measurements at baseline, after 3 months, and after 6 months	Breast Cancer e-Support with four components: a Learning forum, a Discussion forum, an Ask-the-Expert forum, and a Personal Stories forum.	Self-efficacy: Stanford Inventory of Cancer Patient Adjustment; Social support: Multidimensional Scale of Perceived Social Support; Symptom distress: MD Anderson Symptom Inventory; Quality of life: Functional Assessment of Cancer Treatment-Breast; Anxiety and depression: Hospital Anxiety and Depression Scale	Strong
**Survivorship**							
	Ainsworth et al [44] (2018)	To measure time use to further research testing on how to optimize physical activity promotion	N=40	5 days	Time-use measurement app (*Life in a Day*)	Questionnaire assessing functionality and satisfaction with the time use app and Frequency of Forgetting scale	Moderate
	Buscemi et al [48] (2018)	To increase health-related quality of life in Hispanic breast cancer survivors	N=25	4 weeks	Intervention delivered through *MyGuide* app, focused on enhancement of psychosocial adaptation after breast cancer; cancer knowledge; stress awareness and management; social support; and communication with friends, family, and oncology providers. Two to three 15-minute telecoaching calls to facilitate adherence to the *MyGuide* app	Functional Assessment of Cancer Therapy–General Seven Engagement; Acceptability survey; Knowledge about Breast Cancer questionnaire	Moderate
	Fazzino et al [40] (2015)	Weight loss	N=186	6 months	Three-phase intervention: a 6-month weight-loss phase (0-6 months) where all participants received weekly group phone sessions, a 12-month weight-loss maintenance phase (6-18 months) in which participants were randomized to continue group phone sessions or a newsletter comparison condition, and a 6-month no-contact follow-up phase (18-24 months) to evaluate the sustained effects	Qualitative thematic analysis	Included
	Lengacher et al [47] (2018)	To improve psychological and physical symptoms of depression, anxiety, stress, fear of recurrence, cognitive functioning, sleep, fatigue, pain, and quality of life	N=15	6 weeks; Measurements at baseline and at 6‐week follow‐up	The mMBSR(BC)^e^ providing a 2‐hour session intervention weekly for 6 weeks via iPad. Weekly modules on formal meditative techniques (sitting meditation, walking meditation, body scan, and gentle Hatha yoga) via video files and lectures in informal meditative techniques (integrating mindfulness into daily life activities) via audio files	Fatigue Symptom Inventory; Brief Pain Inventory; Pittsburgh Sleep Quality Index; Center for Epidemiological Studies Depression Scale; State‐Trait Anxiety Inventory; Perceived Stress Scale; Concerns about Recurrence Scale; Everyday Cognition; Five Facet Mindfulness Questionnaire; Medical Outcomes Studies Short‐Form; Acceptability and usability ratings	Weak
	Lozano-Lozano et al [45] (2018)	To assess, monitor, and facilitate adherence to healthy lifestyles	N=20	8 days	The BENECA^f^ mHealth app records diet and physical exercise and provides daily notification about energy balance and physical activity and dietary recommendations	Physical activity measured with tri-axial accelerometers; Dietary habits: dietary records and 24-hour dietary recalls	Weak
	McCarroll et al [41] (2015)	Lifestyle program on nutrition quality, physical activity, and eating self-efficacy	N=50	4 weeks	A “beta” health care provider version of the app *LoseIt!* to deliver a comprehensive lifestyle program with emphasis on nutrition quality, physical activity, and eating self-efficacy by a multidisciplinary team that provided real-time feedback notifications	Body mass index; Waist circumference; Anthropometrics; FACT-G^f^; Macronutrient consumption; Physical activity patterns; Weight Efficacy Lifestyle questionnaire	Moderate
	Pope et al [46] (2018)	To improve physiological, psychosocial, and quality of life outcomes	N=10	10 weeks; Measurement at baseline and after intervention	A combined mobile phone app (*MapMyFitness*) and *Facebook*-delivered health behavior change intervention	Physical Activity Readiness Questionnaire; Acceptability survey; Physical activity levels/energy expenditure via Actigraph GT3X^h^ + accelerometer; Weight and body fat percentage; Cardiovascular fitness (YMCA 3-min Step Test); Patient Reported Outcome Measurement Information System; Self-efficacy for exercise scale; Adaptation of the Patient-Centered Assessment and Counseling for Exercise questionnaire; 5-item physical activity enjoyment measure; 14-question measure on physical activity barriers; Outcome expectancy	Weak
	Uhm et al [42] (2016)	To improve physical function and quality of life	Intervention group: N=179; Control group: N=177	12 weeks	Mobile phone exercise app (Smart After Care) and an *InBodyBand* pedometer	International physical activity questionnaire short form; EORTC-QLQ-C30^d^; Quality of Life Questionnaire - Breast Cancer Module 23	Strong
	Valle et al [43] (2017)	Weight gain prevention intervention in African-American breast cancer survivors	Intervention group: N=11; Intervention + activity monitoring group: N=13; Control group: N=11	Baseline, 3 months, and 6 months	Both intervention groups received a face-to-face individual session; a Bluetooth and Wifi-enabled wireless scale with access to a companion mobile app and website with graphs of weight trends; 24 weekly email-delivered behavioral lessons; and 24 weekly emails with tailored feedback on self-weighing and weight data	Weight change and daily self-weighing perception	Strong
	Visser et al [49] (2018)	To provide professional and peer support to breast cancer survivors	Intervention group: N=59; Control group: N=50	3 months Measurements at baseline (1 week prior to the visit) (T0), 1 week (T1), 3 months (T2), and 6 months after the visit (T3)	*My-GMC* intervention: a face-to-face group medical consultation combined with an online app, providing three online support group sessions and additional information	Symptom Checklist-90; Dutch Empowerment Questionnaire for breast cancer patients; Cancer Worry Scale; EORTC-QLQ-C30^d^; EORTC-BR23^g^; Medication Adherence Report Scale; Self-reported usage statistics; QUOTE^h^ questionnaire; Intervention-specific questions	Strong

^a^A detailed table showing features of the extracted studies is available in Multimedia Appendix 1.

^b^The quality of the study was rated using the Effective Public Health Practice Project Quality Assessment Tool for Quantitative Studies [19] and the Joanna Briggs Institute Critical Appraisal Checklist for Qualitative Research [20].

^c^IBIS: International Breast Cancer Intervention Study.

^d^EORTC-QLQ-C30: European Organization for Research and Treatment Cancer Quality of Life Questionnaire - Core 30.

^e^mMBSR(BC): mobile Mindfulness-Based Stress Reduction for Breast Cancer.

^f^FACT-G: Functional Assessment of Cancer Therapy - General.

^g^EORTC-BR23: European Organization for Research and Treatment Cancer Quality Of Life Questionnaire - Breast Cancer Module.

^h^QUOTE: Quality of Care Through the Patient’s Eyes

### mHealth in Breast Cancer Prevention and Early Detection

We identified 7 papers in the area of breast cancer prevention and early detection [22-28]. Hartman et al [22] designed a randomized study to test a weight-loss intervention to reduce the risk of breast cancer. The intervention combined technology-based self-monitoring apps with individualized phone calls. In particular, the intervention entailed an electronic calorie-counting tool, an accelerometer-based activity meter that provides real-time feedback of the number of steps taken and minutes of moderate-intensity activity, and 12 phone calls (30 minutes each) over the 6-month intervention period. After 6 months, the 36 participants in the experimental group had lost significantly more weight and a greater percentage of starting weight than the control group.

*SnapChat*, a social networking mobile app used to deliver educational material on breast cancer through videos, texts, and pictures, increased breast cancer awareness among female students in the Dammam region of Saudi Arabia [23]. Eden et al [24] studied decisional conflict and self-efficacy in 75 women before and after the use of a mammography screening decision aid in the form of an mHealth app and showed that the use of the mHealth app decreased the decisional conflict and increased the decisional self-efficacy. Heo et al [25] developed an mHealth app to encourage breast self-examination. The mHealth app included a breast self-examination reminder, a record-keeping function, and educational features. After using the app, the number of participants who practiced breast self-examination increased from 28 to 32. However, participants below the age of 30 years performed a significantly higher number of breast self-examinations and more appropriate self-examinations than participants aged above 30 years.

An mHealth app developed to convey risk information to patients attending a high-risk breast cancer clinic resulted in increased accuracy in risk perception compared to standard risk counselling [26]. Lee et al [27] tested the efficacy and feasibility of *mMammogram*, an intervention entailing tailored multimedia messages to increase knowledge and awareness and promote mammogram screening among Korean-American immigrant women. The intervention group showed greater knowledge of breast cancer and screening guidelines, readiness for mammography use, and greater satisfaction with the intervention. At the 6-month follow-up, 75% of women who received the *mMammogram* intervention completed the mammograms compared to 30% of the women in the control group. Focus groups with women in the intervention group supported the fact that *mMammogram* enhanced the understanding of breast cancer and screening through *mMammogram* and that health navigators promoted free or low-cost mammograms and scheduling of mammogram appointments [28].

### mHealth and Care Management

A total of 12 papers investigating the use of mHealth apps in breast cancer care management emerged from our review and focused on care management and monitoring of treatment side effects [29-33]; personal data management [34]; psychological aspects, social support, and health-related quality of life [35-38]; physical activity [39]; and educational aspects [12]. The mHealth apps that included the monitoring and care management of treatment side effects covered wound monitoring [30], sleep quality collection [32], daily functional activity stabilization [29], mental health tracker [31], and chemotherapy dose adaptation [33]. These apps were developed for and focused on reliable collection and communication of relevant breast cancer data from patients and oncologists.

In the Hwang’s study [30], a postoperative wound e-monitoring app significantly decreased the number of readmissions to the hospital, unscheduled visits to the emergency department, or walk-in clinic visits. The mHealth app intervention is a virtual care platform that consists of a mobile phone app and secure password-protected online account that allowed patients to take a photo of their wounds postoperatively, attach the photos to electronic messages, and send them to the surgeon using the mobile phone app. The surgeon then responded to each patient’s message within 24 hours. A vast majority (95%) of patients felt that electronic wound monitoring improved their care and would recommend such technologies to a friend or colleague. Min et al [32] analyzed the feasibility of and compliance with an mHealth app for collection of sleep disturbance-related data from breast cancer patients receiving chemotherapy. Participants self-recorded three health experiences—sleep-disturbance symptoms related to mild depression, acute symptoms related to cytotoxic chemotherapeutic agents, and medication diary for antihormonal treatment—that may be caused by breast cancer diagnosis and treatments. The overall compliance during the 90-day longitudinal collection of daily self-reporting of sleep-disturbance data was approximately 45%, confirming the feasibility of the intervention; women without any form of employment exhibited a higher compliance rate. No other association between patient characteristics and compliance was found. An intervention using an mHealth app on patient-reported daily functional activity in both supervised and unsupervised settings showed that the use of the mHealth app in collaboration with physicians was associated with stabilized daily functional activity and fewer and more precise entries than the unsupervised use of the mHealth app. The mHealth and Web app allows patients to record daily functional activity or symptoms with an indication of severity [29]. Kim et al [31] evaluated the validity and screening performance of an mHealth app for depression, which collected self-reported mental health ratings from patients with breast cancer. The app tracked three mental health indicators for depression (sleep satisfaction, mood, and anxiety) daily, and the results strongly supported the potential of a mobile mental health tracker as a tool for screening depression in patients with breast cancer. An mHealth app developed to collect and monitor real-time symptoms in patients receiving oral chemotherapy showed that both patients and oncologists felt reassured by the use of the mHealth app [33]. In the intervention, patients were required to complete a symptom, temperature, and dose diary twice a day using an mHealth app. This information was encrypted and automatically transmitted in real time to a secure server, with moderate levels of toxicity automatically prompting self-care symptom-management messages on the screen of the patient’s mHealth phone or in severe cases, a call from a specialist nurse to advice about care according to an agreed protocol [33].

Klasnja et al [34] tested the feasibility of an mHealth app for data management. The mHealth app included a web component and a mobile component. Using the mHealth app, patients could create, edit, and view the full range of information stored in the website and share them with family members and health care professionals. In addition, patients could create photo, audio, and text notes to quickly capture information and immediately link those notes to related appointments. The mobile app synchronized the app calendar to the phone’s native calendar app, allowing patients see their care events alongside their other commitments. Their study highlighted an empowering effect of mHealth analysis.

Harden et al [39] developed *bWell*, a mobile phone application intervention to optimize self-management of arm and shoulder exercises for upper-limb dysfunction after breast cancer treatment. User testing demonstrated ease of use along with clear, engaging, and motivating content.

Zhu et al explored the effectiveness of *Breast Cancer e-Support*, an app-based program, to address women’s self-efficacy, social support, symptom distress, quality of life, anxiety, and depression [35,36]. The app-based intervention was found to be more effective than usual care in promoting women’s self-efficacy and quality of life and decreasing symptom burden during chemotherapy. The mHealth app did not show any advantage over usual care in terms of social support, symptom severity, anxiety, and depression. In addition, the beneficial effects were not sustained at the 6-month follow-up [36]. Analyzing qualitative interviews, Zhu et al [35] highlighted that participants perceived the program to be helpful in enhancing knowledge, improving confidence level, and promoting emotional well-being. The most prominent benefit was access to tailored advices from experts, whereas the barriers to use were the physical or psychological health status and app instability. Participants suggested implementing message reminders and a search engine to add more interesting and practical knowledge and updating the information more often.

The use of a mindfulness meditation training delivered through a commercially available mindfulness app called *Headspace* that uses audio and animated video improved quality of life and decreased physical and psychological distress [37]. Wallweiner et al [38] analyzed the reliability of a tablet-based measurement app for the European Organization for Research and Treatment Cancer Quality of Life Questionnaire - C30, a measure of health-related quality of life in patients with metastatic breast cancer. The electronic version of this measure was reliable for patients with both adjuvant and metastatic breast cancer, showing a high correlation in almost all questions of the questionnaire.

Foley et al [12] educated patients about breast cancer surgery by using an mHealth app that provided information on the surgery procedure tailored to individual patients. They found that at 7 days after surgery, participants who were more educated about their breast cancer surgery procedure experienced more anxiety than the control participants.

### mHealth in Survivorship

In this review, we identified 10 studies focusing on the use of mobile phone apps among breast cancer survivors. All 10 studies aimed at assessing lifestyle changes, in particular, improving physical activity and reinforcing weight loss [40-46]; reducing stress [47]; improving health-related quality of life [46,48]; and providing information, support, and attention for psychosocial issues [49]. Of the 10 studies, 4 found significant effects of the mHealth apps on weight loss [41-43,46]. McCarroll et al [41] assessed a 1-month lifestyle intervention using a Web and mHealth weight-loss app called *LoseIt!* This intervention placed emphasis on the nutritional quality and physical activity and showed significant reductions in body weight and improvements in eating self-efficacy.

Uhm et al [42] tested and compared a 12-week home-based program of aerobic and resistance exercises using a mobile phone exercise app and a pedometer with a conventional exercise program using a brochure. Physical function, physical activity, and quality of life significantly improved regardless of the intervention method, and changes were not significantly different between the two interventions. The feasibility and preliminary efficacy of two self-regulation interventions using the mHealth app and website as well as tailored feedback emails to prevent weight gain among African-American breast cancer survivors were evaluated [43]. The interventions focused on daily self-weighing and used objective monitoring and tailored feedback about weight and physical activity. Both the interventions were successful in controlling weight, and both intervention groups indicated highly positive perceptions about the intervention; in addition, 100% of participants would recommend the program to other breast cancer survivors. Preliminary findings of an intervention combining the use of a mobile phone app and Facebook-delivered health education material showed an increase in physical activity and decrease in weight and body fat percentage [46]. Fazzino et al [40] performed a thematic qualitative analysis and identified themes related to successful weight loss in breast cancer survivors undertaking a group phone-based weight-loss intervention. Examples of themes that emerged are the importance of being part of a group and internal motivation. *BENECA mHealth*, an application for remotely assessing and monitoring energy balance in breast cancer survivors, showed positive agreement with daily, 24-hour dietary recalls and accelerometer data [45]. Ainsworth et al [44] assessed the acceptability of a 5-day trial of a mobile phone app for measuring time use in order to inform physical activity measurement and promotion interventions. Majority of the participants agreed that learning to use the app was easy, and most preferred to use the app over the paper-and-pencil diary method of record. The 6-week mindfulness-based stress reduction for the breast cancer program delivered via a tablet proved to significantly improve the psychological and physical symptoms of depression, state anxiety, stress, fear of recurrence, sleep quality, fatigue, and quality of life [47]. Buscemi et al [48] pilot-tested a mobile phone-based intervention to boost health-related quality of life in Hispanic breast cancer survivors in the United States. Participants’ knowledge on breast cancer and health-related quality of life increased over the course of the 4-week intervention. A mobile phone app combined with a social media-delivered health behavioral change intervention promoted the improvement of several physiological, psychosocial, and quality of life outcomes over the course of 10 weeks [46]. For example, it improved physical activity and the ability to engage in social roles [46]. The blended care intervention *My-GMC,* which combines a face-to-face group medical consultation with an online app, positively influenced provision of information, support, and attention for psychosocial themes. However, no significant effects on psychological distress and empowerment were observed as compared to usual care [49].

## Discussion

Considering the increasing number of mHealth apps available to patients and their increasing use in breast cancer care, it is important to understand their effects. Therefore, we conducted a systematic review on studies that scientifically tested interventions using an mHealth app for breast cancer care. We identified a total of 29 studies, which encompassed important phases in breast cancer care and addressed prevention and survivorship.

Overall, the results of the studies on the mHealth apps for breast cancer care were promising. Majority of the identified studies in patients’ care management showed a positive impact of the use of mHealth apps. Many hopeful opportunities offered by the mHealth apps rely in the amelioration of the communication process between patient and doctors, favoring an effective exchange of information [33,34]. Positive effects of mHealth-based interventions on health-related quality of life [36,38] and stress reduction [37] have been reported among patients receiving treatment. However, the role of mHealth-based interventions in the psychological impact of treatment is unclear. Only two studies addressed this topic [12,36] but showed no advantage of social support, anxiety, and depression [36] and highlighted an alarming detrimental effect of providing educational material about surgery treatments on patients’ anxiety levels [12].

The number, quality, and findings of studies identified by our search highlight the fact that mHealth technology may play a relevant role in the care of breast cancer survivors. Evidence points to a positive effect of mHealth-based interventions on promoting weight loss [41-43,46], stemming stress, and sustaining the quality of life [44,46,48,49]. However, no convincing data are available on the benefit of mHealth for enduring adverse psychological sequel [46,49]. The restricted number of studies and methodological differences partially account for the heterogeneity of results.

mHealth technology may result in interesting opportunities to improve the lifestyle of individuals at risk of breast cancer and support the spread of knowledge and awareness of breast cancer. However, there is no consistent evidence of the impact of interventions using mHealth apps in breast cancer prevention and early detection due to the limited number of studies identified by our search. Despite the high quality of the studies, only one study considered an mHealth-based intervention in breast cancer prevention [22], and therefore, no strong conclusion can be drawn.

Several aspects have to be considered, as they may limit the generalizability of our results. The heterogeneity in the focus of the included studies may affect the strength of our results and conclusions. Regarding the design and quality of the studies included in our study, a considerable number of studies were feasibility or pilot studies [25,32,33,41,43], which restricted the strength of their results. The data indicate that more evidence is needed to clarify the benefit of mHealth technology for breast cancer care. Examples of the need for more evidence are provided in the studies of Foley et al [12] and Heo et al [25], who found that the effects of their mHealth apps were contrary to what they intuitively expected based on the literature on the effect of education in managing treatment side effects and facilitating adaptation in women with breast cancer [50,51]. Foley et al [12] hypothesized that more information regarding breast cancer would decrease anxiety levels before and after breast cancer surgery. However, their preliminary results showed that women who used the mHealth app experienced higher anxiety levels at 7 days after the surgery than women in the control group. Similarly, Heo et al [25] hypothesized that the use of the mHealth app would augment women’s intentions to regularly perform breast self-examination. This appeared to be true only for women younger than 30 years of age. In contrast, in women older than 30 years, the intention to perform a breast self-examination decreased. A possible explanation of these results may lay in the study design, the sample size, the method used to deliver the program, or the outcomes’ measures.

Despite the aforementioned limitations, the present study shows promising results for the inclusion of mHealth apps in breast cancer care, but also calls for caution when implementing interventions using mHealth technology. The effects of using mHealth apps in the field of breast cancer are only recently explored and may be unpredictable. Few evidence-based interventions are described in the literature, and therefore, there is a need for good-quality clinical studies to guide future implementations.

Our considerations build upon previous evidence highlighting the need for strict regulations in this field and a solid integration between privately tested and research-tested mHealth apps [4,6]. Breast cancer patients should, at all times, be safely assisted with regard to effective management of their health. Therefore, apps need to be extensively research tested before making them available to the public. Scientifically sound data are needed to draw strong conclusions on the utility, effectiveness, and safety of mHealth apps in breast cancer care.

## Appendix

Multimedia Appendix 1 Description of the characteristics of the studies presented in the review.

## Abbreviations

EORTC-BR23: European Organization for Research and Treatment Cancer Quality Of Life Questionnaire - Breast Cancer Module
EORTC-QLQ-C30: European Organization for Research and Treatment Cancer Quality of Life Questionnaire - C30
FACT-G: Functional Assessment of Cancer Therapy: General
IBIS: International Breast Cancer Intervention Study
mMBSR(BC): mobile Mindfulness-Based Stress Reduction for Breast Cancer
mHealth: mobile health
QUOTE: Quality of Care Through the Patient’s Eyes
