# Supplementary material for: Research-Tested Mobile Apps for Breast Cancer Care: Systematic Review
Source: JMIR Mhealth Uhealth. 2019 Feb 11;7(2):e10930. doi: 10.2196/10930 (PMC6388100; doi:10.2196/10930)
Supplement: Multimedia Appendix 1 [file mhealth_v7i2e10930_app1.pdf]

Appendix 1. Description of the characteristics of the studies presented in the review.

Intervention category	Authors and year	Country	Study design	Intervention target	Sample size	Participant characteristics	Duration and follow-up	Intervention components	Outcome measures	Results	Quality of the study[1]
Prevention	Hartman et al. [22] (2016)	USA	RCT	Weight loss	Intervention N=36<br><br>Usual	Women age 40–75 years with increased risk of breast cancer (Gail Model score≥1.7) or previous	6 months	Combined technology-based self-monitoring tools with individualized phone calls	Weight and accelerometer-measured physical activity	At 6 months, intervention participants had lost significantly more weight (4.4 kg VS 0.8 kg, p<0.01) and a	Strong
Early detection	Eden et al. [23] (2015)	USA	Prospective cohort study	To help women in their forties to gain deeper insights into their priorities for screening and prepare them to discuss mammography screening with their health care providers	75	Women age 40–49 years with no known high or moderate risk for breast cancer and no mammography during the previous year	Before and after use of app (same day)	The decision aid (*Mammopad*) included educational modules on breast cancer, mammography, risk assessment, and priority setting about screening	Decisional conflict, Decision Self-efficacy scale	Less decisional conflict, and on all subscales (p<0.01), increased decisional self-efficacy mean scores (p<0.01), different screening intentions (19%) but not significant.	Moderate
	Heo et al. [24] (2013)	Korea	Prospective cohort study	To encourage breast self-examination	45	Women aged ≥19 years with no history of breast cancer and own a smartphone.	Before and after use of app	A smartphone application developed with functions including a breast self-examination date alarm, a reminder to encourage mother and daughter to practice breast self-examination together, record keeping, and educational content with video clips	Survey: increased breast self-examination	An increase from 28 to 32 women who did breast self-examination (p<0.5), subgroups: ≥30 years of age increased from 36,4% to 81.8% (p<0.05), breast self-examination during optimal time increased (2.2% to 33.3%, p<0.01), 8% detected abnormal mass leading to hospital visit	Weak
	Egbring et al. [25] (2016)	Switzerland	RCT	Collection of patient-reported daily functional activity	Controls N=41<br><br>Unsupervised intervention	Women aged ≥18 years, with early breast cancer initiating adjuvant or neoadjuvant chemotherapy and own a smartphone	3 visits	Mobile and Web app to record daily functional activity and adverse events: Patients could report daily functional activity or symptoms with indication of	Functional activity, Eastern cooperative oncology group scoring, common	Daily functional activity declined from the first to third visit for C and the E1(app) group (p<0.05) but not in the E2 (app and physician) (p>0.05), questionnaire	Strong

[1]The quality of the study was rated with the EPHPP Quality Assessment Tool for Quantitative Studies [19] and the Joanna Briggs Institute Critical Appraisal Checklist for Qualitative Research [20].
